# MALAT1 and HOTAIR Long Non-Coding RNAs Play Opposite Role in Estrogen-Mediated Transcriptional Regulation in Prostate Cancer Cells

**DOI:** 10.1038/srep38414

**Published:** 2016-12-06

**Authors:** Aurora Aiello, Lorenza Bacci, Agnese Re, Cristian Ripoli, Francesco Pierconti, Francesco Pinto, Riccardo Masetti, Claudio Grassi, Carlo Gaetano, Pier Francesco Bassi, Alfredo Pontecorvi, Simona Nanni, Antonella Farsetti

**Affiliations:** 1 National Research Council, Institute of Cell Biology and Neurobiology, Rome, 00143, Italy; 2 Università Cattolica, Institute of Medical Pathology, Rome, 00168, Italy; 3 Università Cattolica, Institute of Human Physiology, Rome, 00168, Italy; 4 Università Cattolica, Institute of Pathology, Rome, 00168, Italy; 5 Università Cattolica, Fondazione Policlinico ‘A. Gemelli’, Urological Clinic, Rome, 00168, Italy; 6 Università Cattolica, Multidisciplinary Breast Center, Fondazione Policlinico ‘A. Gemelli’, Rome, 00168, Italy; 7 San Raffaele Pisana Scientific Institute for Research, Hospitalization and Health Care, 00163 Rome, Italy; 8 Goethe University Frankfurt, Division of Cardiovascular Epigenetics, Department of Cardiology, Internal Medicine Clinic III, Frankfurt am Main, 60590, Germany; 9 Goethe University Frankfurt, Internal Medicine Clinic III, Frankfurt am Main, 60590, Germany

## Abstract

In the complex network of nuclear hormone receptors, the long non-coding RNAs (lncRNAs) are emerging as critical determinants of hormone action. Here we investigated the involvement of selected cancer-associated lncRNAs in Estrogen Receptor (ER) signaling. Prior studies by Chromatin Immunoprecipitation (ChIP) Sequencing showed that in prostate cancer cells ERs form a complex with the endothelial nitric oxide synthase (eNOS) and that in turn these complexes associate with chromatin in an estrogen-dependent fashion. Among these associations (peaks) we focused our attention on those proximal to the regulatory region of HOTAIR and MALAT1. These transcripts appeared regulated by estrogens and able to control ERs function by interacting with ERα/ERβ as indicated by RNA-ChIP. Further studies performed by ChIRP revealed that in unstimulated condition, HOTAIR and MALAT1 were present on pS2, hTERT and HOTAIR promoters at the ERE/eNOS peaks. Interestingly, upon treatment with17β-estradiol HOTAIR recruitment to chromatin increased significantly while that of MALAT1 was reduced, suggesting an opposite regulation and function for these lncRNAs. Similar results were obtained in cells and in an *ex vivo* prostate organotypic slice cultures. Overall, our data provide evidence of a crosstalk between lncRNAs, estrogens and estrogen receptors in prostate cancer with important consequences on gene expression regulation.

Hormone Nuclear Receptors (NRs) are ligand-dependent transcription factors critically involved in a plethora of physiological processes including growth, differentiation, homeostasis, development and metabolism (reviewed in refs 1, 2, 3). Conversely, deregulated NRs participate in a range of pathophysiological processes such as diabetes, hormone resistance syndrome and cancer. Chromatin immunoprecipitation coupled with massively parallel DNA Sequencing (ChIP-Seq) has allowed detailed mapping of NR-binding sites across the whole genome, revealing a strong preference for hormone NRs to bind genomic regions that are distal from transcription start sites of coding genes (>10 kb)^4^,^5^. Moreover, it has been shown that multiple NRs can bind to the same genomic locus through cooperative or antagonistic interactions^6^,^7^,^8^ with components of the nitric oxide- or hypoxia-mediated signaling pathways.

Distinct long non-coding RNAs (lncRNAs) have been recently found to play roles in human diseases, including cancer (in ref. 9 and references therein) and have been proposed to affect hormone nuclear function (in ref. 10 and references therein). Aberrant expression of lncRNAs has been linked to cancer progression and an altered chromatin state promoting metastasization, both independent predictors of patient outcome^11^,^12^,^13^,^14^,^15^. Despite the growing number of annotated lncRNAs, relatively little is known about their differential regulation as compared to coding genes, although it appears clear that they are under distinct regulatory regimens, contain specific transcription factor binding sites and exhibit a unique pattern of chromatin marks^16^,^17^.

Our prior work identified the endothelial nitric oxide synthase (eNOS) as an active component of the estrogen receptor complex with a role in telomerase transcription^6^,^18^. We found that, in primary and metastatic prostate cancer (PCa) cells, a consistent number of eNOS-DNA bound complexes formed in an estrogen-dependent fashion along the regulatory genomic regions containing Estrogen Response Elements (EREs) of specific noncoding RNAs associated with cancer (e.g. miR34a)^19^. Here, we queried how lncRNAs aberrantly expressed in hormone-driven tumors, including HOX transcript antisense RNA (HOTAIR), Metastasis Associated Lung Adenocarcinoma Transcript 1 (MALAT1), and ANRIL (as known as CDKN2B-AS1), regulate estrogen receptor signaling (reviewed in ref. 20 and references therein). Specifically, we report about the molecular dissection of the mechanisms involved in the interaction between HOTAIR, MALAT-1 and ERs/eNOS on specific chromatin regions in PCa cells upon 17β-estradiol stimulation.

## Results

### Cancer-associated lncRNAs as estrogen transcriptional targets in prostate and breast cancer cells

Our published prostate cancer cell ChIP-seq database^19^ revealed widespread localization of the eNOS complex on non coding genomic regions. Here, we focused on HOTAIR, MALAT1 and ANRIL lncRNAs, whose aberrant expression has been found to correlate with progression in a variety of human cancers including hormone-driven tumors^11^,^12^,^13^.

**Figure 1:**
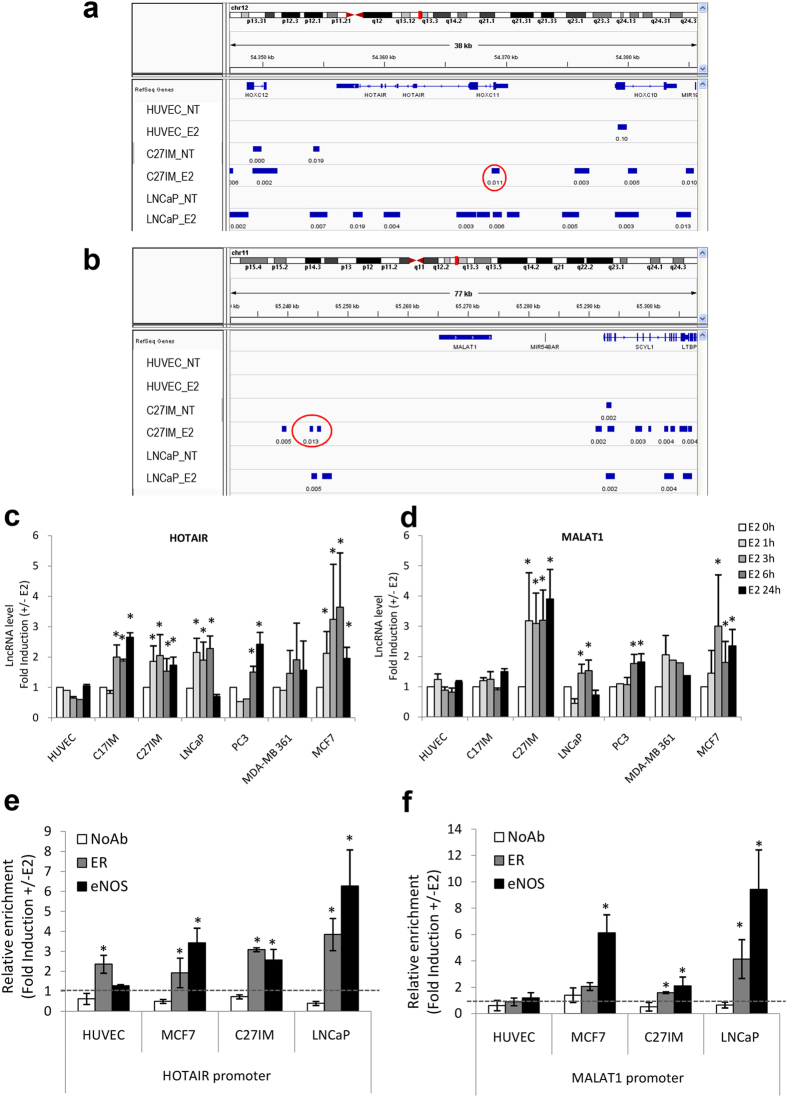
Schematic representation of the peaks of eNOS by ChIP–Seq, estrogen-dependent LncRNAs expression and eNOS/ERs recruitment in prostate and breast cancer cells. (**a**,**b**) Integrated Genome Viewer (IGV 2.3) screenshots showing peaks of eNOS identified by ChIP–Seq at the genomic regions encoding HOTAIR and MALAT1 in two prostate cancer cell lines, LNCaP and C27IM, and in the human endothelial cell line HUVEC, in the absence (NT) or presence of 17β-estradiol (10 nM E2). Region amplified in panel e and f are indicated as red circles. (**c**,**d**) Quantification of HOTAIR and MALAT1 expression by qRT-PCR in normal HUVEC, prostate hyperplastic C17IM, BCa (MCF7, MDA-MB 361) and PCa (C27IM, LNCaP, PC3) cells in basal condition (E2 0 h) and after 3 h, 6 h and 24 h of treatment with E2. The results are plotted as fold induction (+/−E2) and represent the average of 5 independent experiments. *p < 0,05 vs E2 0 h. (**e**,**f**) Recruitment of Estrogen Receptors (ER) and eNOS, on the promoter region of HOTAIR and MALAT1 by ChIPs in the presence or absence of E2 in HUVEC, breast (MCF7) and prostate (C27IM and LNCaP) cells. The immunoprecipitations were performed using anti ERα (in HUVEC and MCF7), ERβ (in C27IM and LNCaP) and eNOS or no antibody (NoAb) as a negative control. Values are represented as Fold of Induction (+/−E2) and as mean +/−SEM of 4 independent experiments. *p < 0,05 +E2 vs −E2.

Analysis of these lncRNAs by ChIP-Seq in prostate cancer cells showed that the eNOS-peaks were present on the HOTAIR and ANRIL promoter region before and after stimulation with 17β-estradiol (E2) and on the MALAT1 promoter region exclusively upon E2 treatment (Fig. 1a,b and Supplementary Fig. S1). Interestingly, the eNOS-bound complexes were virtually absent along other lncRNAs mostly associated with PCa, such as SCHLAP1, PCA3, PCAT1, PGCEM1 and PRNCR1 (Supplementary Fig. S1). In addition, eNOS peaks overlapping with the regulatory regions (−20 kb from TSS) of HOTAIR, MALAT1 and ANRIL lncRNAs were present in PCa (C27IM and LNCaP) but absent in human umbilical vein endothelial (HUVEC) cells (in ref. 19 and Nanni *et al*. *manuscript in preparation*).

Expression of lncRNAs before and after estrogen stimulation was carried out on breast and prostate cancer cell lines, both derived from hormone-responsive tumors, as well as in human primary endothelial cells (HUVEC) used as reference. In all cases, the expression of endogenous estrogen receptors α and β (ERα, ERβ), androgen receptor (AR), and eNOS was assessed by Western blot (*not shown*). Quantitative Real Time PCR (qRT-PCR) revealed that, in the unstimulated condition, tumor cells expressed variable levels of HOTAIR, MALAT1 and ANRIL (Supplementary Fig. S2). We also evaluated the SCHLAP1 transcript, known to promote invasion and metastasis^21^, which exhibited a very high and selective level of expression in metastatic PCa cells. The lncRNA GAS5, which has been shown to act as a tumor suppressor^22^, was used as control. As expected, GAS5 transcript was abundant in HUVEC and normal breast epithelial cells (MCF10 cell line, *data not shown*), but barely detectable in all tested breast and prostate cancer cells.

To assess the estrogen responsiveness of the selected lncRNAs associated with the eNOS-peaks (in ref. 19 and Fig. 1a and b), cell lines representative of prostate or breast cancer were exposed to E2 (10 nM) for 1, 3, 6 or 24 hours. Upon E2, a significant increase of HOTAIR and MALAT1 transcripts was observed in breast cancer, PCa and benign prostate hyperplasia (C17IM) cells. ANRIL exhibited an overall weaker estrogen responsiveness compared to HOTAIR and MALAT1 and was not included in further experiments. In agreement with ChIP-Seq data, none of the tested lncRNAs has increased expression in HUVEC (Fig. 1c,d and Supplementary Fig. S3).

Of note, the SCHLAP1 transcript was not modulated by estrogen (E2, 10 nM) or androgen (dihydrotestosterone, DHT, 10 nM) treatment (Supplementary Fig. S3).

### Role of eNOS and estrogen receptors in the hormonal regulation of HOTAIR and MALAT1

Next, we investigated the direct involvement of eNOS and/or ERs in HOTAIR, and MALAT1 transcriptional regulation. As reported in previous studies from our group, in response to estrogenic stimuli, eNOS and ERs form nuclear complexes that modulate gene transcription in HUVEC and prostate cancer cell lines^6^,^18^,^19^,^23^. Validation of the eNOS peaks within HOTAIR and MALAT1 regulatory regions as emerged by ChIP-Seq was confirmed by traditional ChIP in HUVEC, C27IM, and LNCaP cells in independent experiments (Fig. 1e and f). ERα-positive MCF7 breast cancer cells were included as hormone responsive control cell line. Results showed estrogen-dependent recruitment of eNOS and ERs on the HOTAIR and MALAT1 promoter region at the eNOS peaks in endothelial, breast cancer cells, and PCa cells. No estrogen-dependent promoter occupancy by eNOS and ERs was observed in the SCHLAP1 genomic region encompassing TSS (Supplementary Fig. S3c).

In both PCa and breast cancer cells 45 min of estrogen treatment was sufficient to induce a consistent increase of eNOS recruitment onto the HOTAIR and MALAT1 promoter regions as compared to basal condition. In agreement with our previous study^6^, recruitment of eNOS was paralleled by that of ERβ in PCa cells and of ERα in breast cancer cells. In HUVEC, with the exception of ERα on the HOTAIR promoter, no substantial eNOS or ERβ recruitment was observed, in agreement with ChIP-Seq data (Fig. 1e and f). These results suggest that hormonal regulation of both lncRNAs may occur through the formation of complexes containing ERα or ERβ and eNOS in cancer but not in normal endothelial cells.

In line with this, eNOS silencing by antisense oligos or the competitive inhibitor 7 Nitroindazole (7N) efficiently abrogated HOTAIR estrogen responsiveness in PCa cells (Supplementary Fig. S3).

### HOTAIR and MALAT1 associate to ERα/β and/or eNOS on chromatin in estrogen-dependent fashion

**Figure 2:**
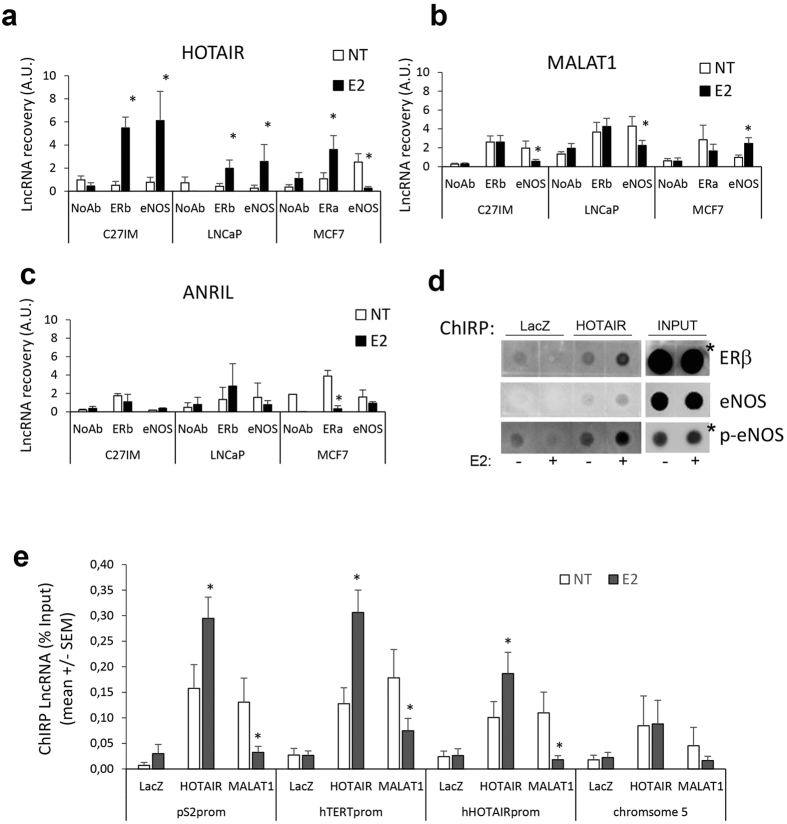
*In vivo* analysis of HOTAIR, MALAT1 or ANRIL interaction with ERs and eNOS and recruitment on estrogen–target genes promoter before and after estrogen treatment. (**a**–**c**) *In vivo* HOTAIR (**a**), MALAT1 (**b**) or ANRIL (**c**) interaction with ERs or eNOS before and after 17β-estradiol (10 nM E2, 45 min) detected by RNA-ChIP assays. RNA-ChIPs were performed using antibodies specific to ERbeta (ERb), in PCa, primary tumor- or metastatic-derived cells, C27IM and LNCaP respectively, or to ERalpha (ERa), in MCF7, or to eNOS or in the absence of Ab (NoAb) as negative control. Immunoprecipitated RNA was recovered and analysed by qRT-PCR. Results are mean +/−SEM of 5 independent experiment. *p < 0.05 E2 vs NT. (**d**) *In vivo* interaction between ERb or eNOS with HOTAIR was detected in PCa (LNCaP) cells before and after 17β-estradiol (10 nM E2, 45 min) by ChIRP assays. ChIRP was performed using DNA antisense oligos specific for HOTAIR or LacZ as negative control. Protein-bound were recovered and analysed by Dot Blot with antibodies specific for ERb or total eNOS or phosphorylated form of eNOS (p-eNOS). White lines indicate samples spotted in noncontiguous lanes (*lower exposure). (**e**) Recruitment of HOTAIR and MALAT1 on the promoter region of estrogen target genes (pS2, hTERT and HOTAIR) or region without eNOS-peak (chromosome 5) by ChIRPs in the presence or absence of estrogen (10 nM E2). LacZ served as a negative control. Values are calculated % Input and data are represented as mean +/−SEM of 5 independent experiments. *p < 0.05 E2 vs NT.

The potential association between HOTAIR or MALAT1 and ERs and/or eNOS before and after E2 treatment was investigated by RNA-Chromatin Immunoprecipitation (RNA-ChIP) (Fig. 2a–c).

Results showed that association between HOTAIR and ERβ occurred upon estrogen treatment of the primary prostate tumor-derived C27IM cells and PCa metastasis-derived LNCaP. A similar trend was observed for HOTAIR and ERα in MCF7.

Notably, association between HOTAIR and eNOS, which was virtually absent in basal condition, occurred in C27IM and LNCaP upon estrogen treatment. However, in breast cancer cells, this interaction was detectable in basal condition and abolished upon E2 administration.

RNA-ChIP with MALAT1 (Fig. 2b) revealed a differential response: interaction between MALAT1 and ERβ in PCa cells and or between MALAT1 and ERα in breast cancer cells, both detectable in basal condition, was not affected by E2 treatment. MALAT1 association with eNOS was significantly reduced upon E2 in C27IM and LNCaP, whereas it was enhanced in MCF7 cells.

No appreciable association between ANRIL and ERs or eNOS was detected by RNA-ChIP in either prostate or breast tumor cells (Fig. 2c), with the exception of ERα in MCF7 cells, where interaction with ANRIL was present in the unstimulated condition and abrogated upon E2.

By a reciprocal approach, HOTAIR and MALAT1 interaction with ERβ and/or eNOS was also evaluated in LNCaP by Chromatin Isolation by RNA Purification (ChIRP) (Fig. 2d). Precipitation of crosslinked chromatin with specific antisense oligos for HOTAIR or MALAT1 was performed and protein-bound complexes recovered and assessed by Dot Blot. LacZ served as negative control. HOTAIR/ERβ and HOTAIR/eNOS or HOTAIR/phospho-eNOS association was nicely induced by E2 treatment. MALAT1 interaction with ERβ was detectable in basal condition and remained substantially unchanged upon estrogen treatment. No apparent interaction between MALAT1 and eNOS was detected (*data not shown*).

Next, ChIRP assay specifically designed to identify sites of RNA-Chromatin interaction and to map lncRNAs occupancy before and after estradiol treatment, showed consistent occupancy by HOTAIR and MALAT1 of promoters of estrogen target genes such as pS2 (also known as TFF1), hTERT and HOTAIR itself at the eNOS peak/EREs sites (Fig. 2e). Interestingly, the estrogen treatment considerably enhanced HOTAIR while inhibited MALAT1 recruitment onto all three promoters analyzed. As expected, no interaction was observed in a chromosome 5 region assessed as negative for EREs or eNOS-peaks in prior ChIP-Seq analysis^19^.

Taken together, these results are suggestive of an opposite role for HOTAIR and MALAT1 on chromatin, specifically a positive role for HOTAIR, whose recruitment was indeed strengthened upon estrogen treatment, and a repressive role for MALAT1, which detached from specific genomic regions in a tight estrogen-dependent fashion.

### HOTAIR positively regulates estrogen-target genes by estrogen-modulated chromatin remodeling

**Figure 3:**
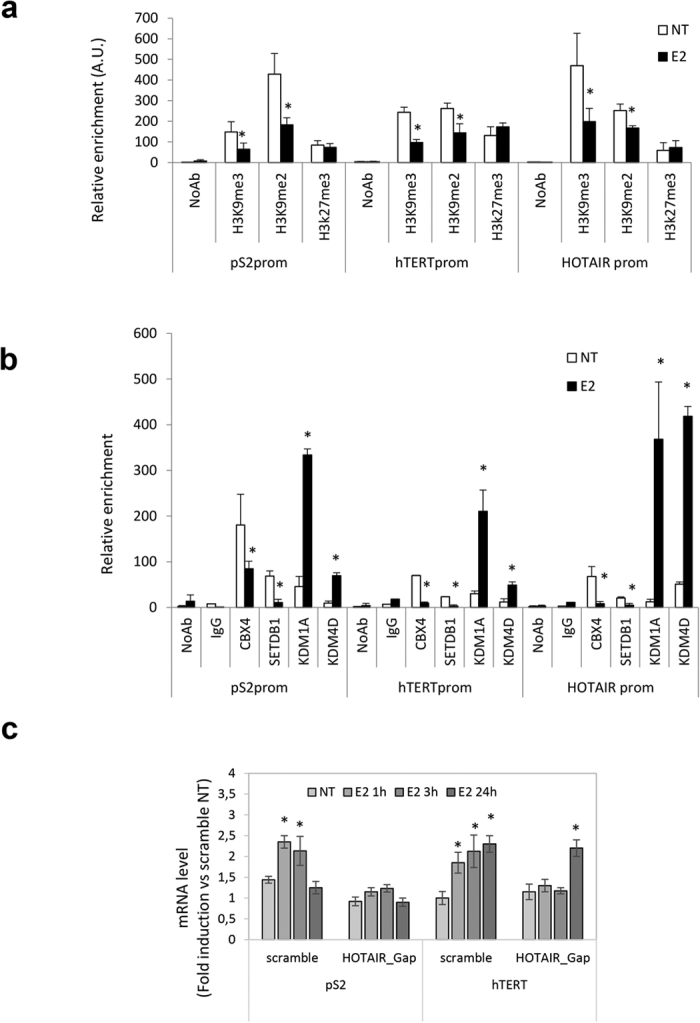
ChIPs analysis of histone tails and methylases/demethylases recruitment modification on estrogen-target genes promoter before and after estrogen treatment and effect of interference for HOTAIR on estrogen responsiveness. (**a**) Recruitment of H3K9me3, H3K9me2 and H3K27me3 on pS2, hTERT and HOTAIR promoters by ChIPs in PCa cells in the presence or absence of estrogen (E2). No Antibody (NoAb) served as negative control. Data are represented as relative enrichment in Arbitrary Unit (A.U.) of 5 independent experiments. *p < 0,05 E2 vs NT. (**b**) Recruitment of transcriptional suppressor CBX4, methylases SETDB1, demethylases KDM1A (LSD1) and KDM4D (JMJD2D) on promoters as in **a**. No Antibody (NoAb) or IgG served as negative control. Data are represented as relative enrichment in Arbitrary Unit (A.U.) of 5 independent experiments. (**c**) pS2 and hTERT mRNA levels quantified by qRT-PCR in LNCaP cells transfected with HOTAIR or scramble gapmers, in basal condition (NT) and after 10 nM 17β-Estradiol treatment of 1, 3 or 24 hours. The results are plotted as fold induction vs “scramble_NT” +/−SEM of 5 independent experiments. *p < 0.05 E2 vs NT.

To investigate the role of HOTAIR as positive regulator of estrogen receptor-regulated transcription, we first excluded the involvement of the EZH2 Polycomb Repressive Complex 2 (PRC2) subunit, a known interactor of HOTAIR. ChIP assays for EZH2 and the associated modification H3K27me3, directly mediated by PRC2^24^,^25^, clearly showed that estrogen treatment did not induce EZH2 recruitment or H3K27me3 at the promoter of estrogen-induced target genes (Fig. 3a and *data not shown*), in agreement with the observed transcriptional activation and with previous study reporting HOTAIR occupancy regardless of EZH2^26^.

Next, we investigated the potential involvement of enzymes such as Lysine (K)-Specific Demethylase 1 A (KDM1A, also known as LSD1), known to be a HOTAIR partner^27^ and Lysine (K)-Specific Demethylase 4D (KDM4D, also known as JumonjiC (JmjC)-domain-containing demethylase JMJD2D)^28^. The KDM1A is capable of repressing promoter activity by demethylation of Histone H3 mono/dimethyl Lys4 and of activating transcription through the demethylation of Histone H3 mono/dimethyl Lys9^29^,^30^. KDM4D was also considered because of its RNA-dependent chromatin localization strictly affecting the levels of Histone H3 Lys9 tri/demethylation^28^. In this respect, we hypothesized that HOTAIR recruitment at eNOS-peaks and EREs on estrogen target genes after E2 stimulation (Fig. 2e) modulates level of H3K9me3 and H3K9me2 by affecting binding to chromatin of KDM1A and KDM4D and/or methyltransferase SET domain bifurcated 1 (SETDB1). Recruitment of KDM1A and KDM4D as well as H3K9me3 and H3K9me2 modifications were analyzed by ChIP (Fig. 3a and b). Of note, a specific estrogen-dependent recruitment of both demethylases KDM1A and KDM4D was observed at the ERE site/eNOS peaks of the estrogen target genes. The hormonal-dependent occupancy at these sites by both demethylases was paralleled by a decrease in histone Lysine 9 methylation, including H3K9me3 and H3K9me2. Consistent with this, estrogen-dependent detachment of methyltransferase SETDB1 inversely correlated with recruitment of KDM1A and KDM4D demethylases.

To gain deeper insight into HOTAIR function in ER signaling, we next evaluated the consequences of HOTAIR depletion by gapmers on the estrogen responsiveness in prostate cancer cells transfected with specific or scramble oligonucleotides. HOTAIR depletion about 50% efficiency (Supplementary Fig. S4), resulted in abrogation (pS2) or delay (hTERT) of the estrogen response; in the latter case, 24 h exposure to E2 was necessary, upon HOTAIR interference, to achieve about 2.5-fold increase of hTERT mRNA (observed after 3 hours upon scramble oligos; Fig. 3c). Of note, responsiveness to ERβ selective ligand, 3βAdiol, was abrogated by both HOTAIR and MALAT1 knockdown (Supplementary Fig. S4), indicating that both lncRNAs are necessary to achieve a complete estrogen responsiveness upon treatment with this androgen metabolite capable to selectively bind ERβ^23^,^31^. Similarly, an abrogation of pS2 estrogen sensitivity was observed by interference of MALAT1 (efficiency about 80%, Supplementary Fig. S4) in both primary tumor-derived (C27IM) and metastatic cells (LNCaP) (*data not shown*).

Interestingly, transfection with specific HOTAIR or MALAT1 gapmers did not impair responsiveness of a classical androgen target gene, PSA, to DHT (Supplementary Fig. S4c).

These data indicate that MALAT1 and HOTAIR appear to control estrogen receptor but not androgen receptor-activated pathways.

### MALAT1 as repressor of gene transcription

**Figure 4:**
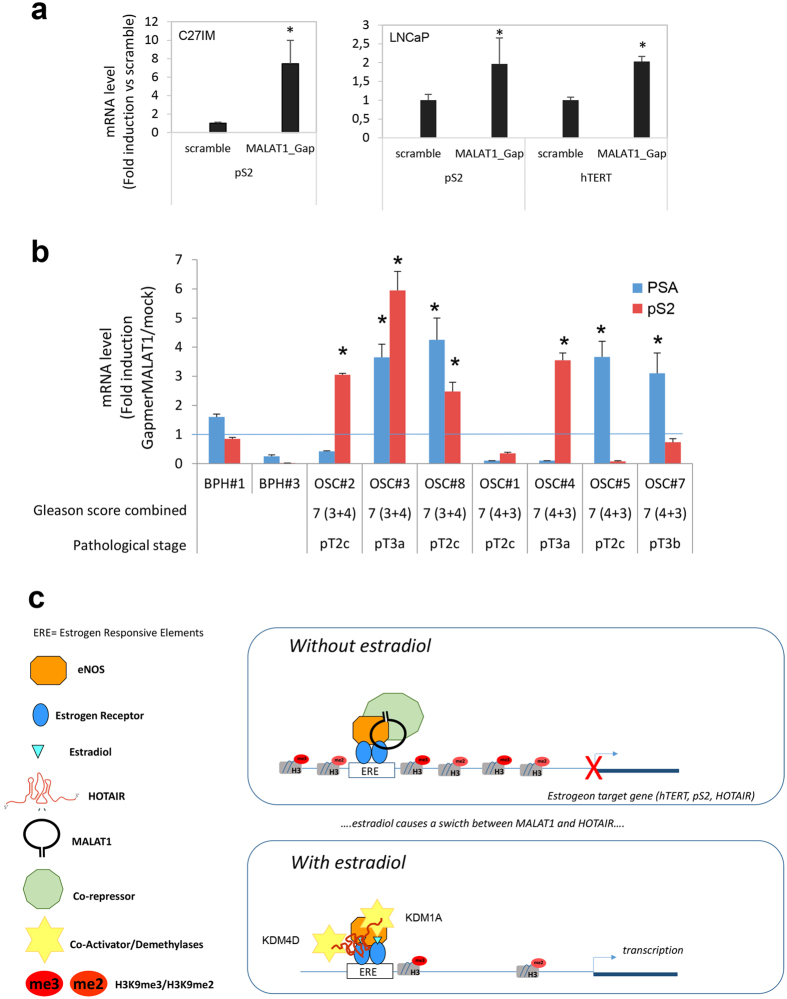
Effect of interference for MALAT1 on estrogen responsiveness in PCa cells and organotypic slice cultures of Prostate Tumors and model for HOTAIR and MALAT1 function in estrogen signaling. (**a**) hTERT and pS2 mRNA levels in PCa cells (C27IM, left, and LNCaP, right) quantified by qRT-PCR in PCa cells transfected with MALAT1 or scramble gapmers. The results are plotted as fold induction vs scramble +/−SEM of 5 independent experiments. *p < 0.05 vs scramble. (**b**) pS2 and PSA mRNA levels quantified by qRT-PCR PCa-organotypic slices cultures (OSC) transfected with MALAT1 Gapmer for 4 days. Data represent results of 7 different PCa-patients and 2 Hyperplasia (BPH) and are plotted as Fold induction vs mock (mean +/−SEM, *p < 0,05 vs mock). (**c**) Role of HOTAIR and MALAT1 in the ER/eNOS dependent signaling on estrogen-responsive genes in aggressive Prostate Cancer.

In agreement with ChIRPs data, a repressive role for MALAT1 was unmasked by MALAT1-antisense experiments. We analyzed the consequences of MALAT1 depletion on basal transcription of a subset of hormone-sensitive genes such as pS2, hTERT and PSA. Depletion of MALAT1 augmented pS2 and hTERT mRNAs basal expression, in both C27IM and LNCaP cells (Fig. 4a). To corroborate this finding we asked whether similar de-repression could be observed in Organotypic Slice Cultures (OSCs) of organ-confined prostate tumors obtained from fresh surgical explants (Supplementary Fig. S5). Remarkably, MALAT1 depletion (about 40%, Supplementary Fig. S5) determined a significant increase in the pS2 and PSA basal transcription (ranging from 2.5 to 7-fold induction vs scramble), thus validating *in vivo* our finding of the repressive function played by MALAT1 in PCa (Fig. 4b and Table 1).

Other studies have shown direct interaction between MALAT1 and Polycomb Repressive Complex 1 (PRC1) subunit CBX4^32^, and an estrogen-independent genomic localization of MALAT1 in MCF7 cells^33^. We used ChIP assays to analyze occupancy by CBX4 of three estrogen-regulated gene promoters, pS2, hTERT and HOTAIR. In line with our assumption, a consistent reduction of CBX4 recruitment on all three promoters paralleled by a decrease in occupancy by the methyltrasferase SETDB1 on the same regulatory genomic loci was observed (Fig. 3b).

We next asked whether MALAT1 or HOTAIR may associate with ERβ or eNOS forming combinatorial complexes on chromatin. Serial RNA-Re-ChIP experiments showed results consistent with the RNA-ChIP and ChIRP assays (Fig. 2), in the same experimental conditions (Supplementary Fig. S5f). Specifically, HOTAIR association with eNOS/ERβ complex occurred exclusively upon E2 stimulation, whereas that of MALAT1 exhibited a trend towards reduction and a net decrease when antibody to eNOS immunoprecipitation preceded ERβ immunoprecipitation (eNOS:ERβ). Again no association was observed between ANRIL and ERβ or eNOS, alone or in combination, regardless of estrogen treatment, substantiating the finding of an unprecedented estrogen-modulated association between HOTAIR or MALAT1 with the ERβ/eNOS combinatorial complex on chromatin.

## Discussion

Intensive research has been devoted nowadays to the molecular dissection of lncRNAs serving as signals of cellular programs active in cancer by providing prognostic value or even foreseeable therapeutic options for patients^34^. Our study contributes to this concept by demonstrating that HOTAIR and MALAT1 not only are estrogen receptor transcriptional targets but also act as ERs partners in terms of their capability to regulate the estrogen-dependent and independent transcription of genes such as pS2, hTERT and PSA, *in vitro* and *in vivo*.

The driving hypothesis envisaging a key role for HOTAIR and MALAT1 as relevant players of hormone action stems from our previous work and from recent data from the literature^10^. The estrogen-regulated pattern of eNOS-containing complexes present along the regulatory genomic regions of a subgroup of cancer-associated lncRNAs, specifically HOTAIR and MALAT1, prompted us to investigate them as potential targets as well as effectors of the ER signaling in a non-canonical microenvironment such as that of prostate cancer. Compelling experimental data support a critical yet unresolved role of the estrogen receptor in PCa^6^,^19^,^23^,^35^,^36^,^37^, mediated by its participation in a multiprotein complex containing eNOS and/or HIFs, along the regulatory genomic regions of a subset of genes associated with poor prognosis such as VEGF, Glut-1 and others.

Our findings of HOTAIR and MALAT1 association with both ERα/ERβ detectable at chromatin level by RNA-ChIP and ChIRP is suggestive of a novel molecular mechanism expanding our knowledge of hormone action. Their interaction with ERs but also with eNOS appears in fact to be necessary for a complete estrogen responsiveness, as indicated by HOTAIR and MALAT1 depletion experiments showing abrogation of pS2 estrogen sensitivity both *in vitro* and *in vivo*.

Notably, the mechanism adopted by HOTAIR and MALAT1 to achieve abrogation of the hormone response seems substantially different. Upon HOTAIR depletion we found in fact loss or delay of the E2-mediated induction above basal transcription of specific target genes (Fig. 3c), whereas upon MALAT1 knockdown, the estrogen responsiveness was essentially blunted because of the increase in the basal transcription of target genes (Supplementary Fig. S5e and *data not shown*). This different mode of action was substantiated also by ChIP data with antibodies to specific demethylases/methyltransferases or PRC1 complex subunit CBX4 along three different gene target promoters (Fig. 3). Transcriptional regulation of estrogen-target genes requires, at least in the prostate cancer cell microenvironment, an estrogen-dependent chromatin remodeling involving demethylases or methyltransferases or CBX4.

Thus our experimental data demonstrate that both lncRNAs may function as chromatin modifiers affecting transcription in a hormone-dependent and independent fashion. The novelty of our work resides in the identification of a new estrogen-dependent association between HOTAIR/MALAT1 and ERβ/ERα (in prostate or breast cancer cells, respectively) and/or eNOS. The latter has been already identified in our previous studies as cofactor of both ERα and ERβ^6^,^18^,^19^,^23^ in normal human endothelial or prostate cancer cells. Given the key role played by eNOS in the progression of PCa, it is conceivable that the association HOTAIR/eNOS and the HOTAIR/ERβ/eNOS complex on chromatin (as assessed by RNA-Re-ChIP, Supplementary Fig. S5f) appears to be an essential molecular feature associated with advanced prostate cancer. Whether the association between HOTAIR and eNOS in the breast cancer environment, detectable in the basal condition, is instead abrogated upon E2 because of the presence of endogenous high level of ERα (absent in C27IM primary tumor-derived cancer cells) is intriguing and requires further investigations.

Of translational relevance, we showed evidence of a potential repressive function of MALAT1 on the basal transcription of sex steroid hormone receptors target genes such as pS2 and PSA in primary prostate cancer cells and *ex vivo* organotypic slice cultures derived from PCa patients. Our finding represents a proof of principle of a putative “response” upon MALAT1 silencing of two well characterized hormone-dependent genes relevant for prostate cancer, disclosing potential perspective manipulations in terms of transcription regulation of prognostic genes.

Based on our results the following model can be drawn (Fig. 4c): in the absence of estradiol the ER/eNOS complex is recruited at Estrogen Response Elements together with MALAT1 and a Co-repressor (putatively CBX4) on the regulatory region of target genes. This event facilitates a close chromatin conformation, resulting in repression of transcription. On the contrary, upon estradiol, both MALAT1 and Co-repressor detach from the ER/eNOS complex while HOTAIR binds to chromatin eventually making contacts with demethylases KDM1A and KDM4D at eNOS-peaks on estrogen-target promoters, likely switching histone marks from repressive to active status.

Overall our finding supports the concept that HOTAIR and MALAT1 estrogen-responsiveness and their control of hormone-sensitive genes may be critical for pharmacological targeting of lncRNAs.

## Materials and Methods

### Antibodies

The following antibodies were used: anti-ERβ (L-20), anti-ERα (HC-20) and anti-AR (C-19) from Santa Cruz Biotechnology, Dallas US-TX; anti-ERβ from GeneTex, Irvine, CA; anti-eNOS from BD Biosciences, Franklin Lakes, US-NJ; anti-phospo-eNOS (Ser1177) from Cell Signaling, Danvers, US-MA; anti-AR (PG-21) from Upstate, Lake Placid, US-NY; anti-ERα Ab-10 (TE111.5D11) from Thermo Fisher Scientific, Fremont, US-CA; anti-KDM1A (also named LSD1), anti-H3K9me3, anti-H3K9me2 and anti-H3K27me3 from Active Motif, Carlsbad, US-CA; anti-H3K9me2 from Epigentek, Farmingdale, US-NY; anti-CBX4 (also named Pc2), anti-KDM1A and anti-SETDB1 from Bethyl Laboratories, Montgomery US-TX; anti-KDM4D (also named JMJD2D) from Novus Biological, Littleton US-CO; anti-EZH2 from Abcam, Cambridge UK; anti-HSP70 from StressGen Biotechnologies, San Diego US-CA.

### Cell cultures, treatments and eNOS interference

HUVEC and PCa cells cultures and treatments were as in refs 6, 18, 23, 36 and 38. ATCC Breast cancer (BCa) cells were grown in DMEM, with the exception of MDA-MB-361 grown in RPMI 1640. All media were supplemented with 10% fetal bovine serum, 2 mM glutamine, 100 µg/ml penicillin and streptomycin. At least 72 h before experimental use, cells were switched to a medium with hormone-deprived serum and treated with 17β-estradiol (E2), 5α-Androstane-3α, 17 α-diol (3β-adiol), 5 α-Androstan-17 α-ol-3-one (DHT) (Sigma-Aldrich, St. Louis, MO) or 7-nitroindazole (7N) (Biomol, Farmingdale, NY), for the concentrations and the times indicated in the figure legends. Small interference to eNOS was obtained with 100 nM Trilencer-27mer siRNA duplexes (Origene, Rockville, MD) transfected by Lipofectamine RNAiMAX according to the manufacturer’s instructions in hormone-deprived serum and after 72 h treated whit 17β-estradiol (E2).

### Western and Dot blot assay

Western blotting analysis was performed as in ref. 19. Evaluation of eluted/precipitated proteins after ChIRP was performed as described by ref. 26. Briefly 1% SDS and Laemmli buffer were added to sample before boiling for 5 min and running on 7,5% SDS-PAGE gel. For Dot Blot, 1% SDS was added and samples blotted to nitrocellulose membrane.

### RNA extraction and qRT-PCR analysis

RNA isolation and cDNA preparation was as described in refs 6 and 36. Real-time PCR was performed three times in duplicate on an ABI Prism 7500 Detection System and/or QuantStudio 5 Real-Time PCR System (Applied Biosystems). Relative amount of each lncRNAs was measured as fold change using the 2-ΔΔCt method (βActin or GAPDH served as endogenous control).

Primers sequences are as in ref. 6 and as follows:

HOTAIR 5′-CTCCAGGCCCTGCCTTCT-3′ and 5′-AGCACAGGCGAGTCAGAGTTC-3′;

MALAT1 5′-GCAGGCGTTGTGCGTAGAG-3′ and 5′-TTGCCGACCTCACGGATT-3′;

ANRIL 5′-GAGAGGGTTCAAGCATCACTGTT-3′ and 5′-TCTCCCCGGTTTTCTTCTAGAA-3′:

GAS5 5′-CCATTGGCACACAGGCATTA-3′ and 5′-GGCAAGTTGGACTCCACCAT-3′;

SCHLAP1 5′-CCAGTTCTGGACACAATTTCAAGT-3′ and 5′-GGTTGAATAAAAATCACTGCCATTT-3′;

PSA 5′-GTGGGTCCCGGTTGTCTTC-3′ and 5′-CCACAATCCGAGACAGGATGA-3′.

### ChIPs

Chromatin cross-linking and ChIP assays (n = 5) were performed as described^6^, using antibodies specific as indicated in figures and legends. Negative controls were generated omitting antibody or using Ab to IgG (NoAb or IgG). DNA fragments were recovered and analyzed by qPCR as previously described^19^. Briefly, qPCR was performed in duplicate or triplicate and the data, normalized to the corresponding DNA input control, are represented as relative enrichment. Primers sequences for promoters, designed using Primer express3.0 or 3.1 (Applied Biosystems), are as in ref. 19 (hTERT prom, pS2 and empty-region Chromosome 5) and as follows:

hHOTAIRprom-eNOSpeak 5′-GCTGCAGTATTTCTCGGGAAA-3′ and 5′-CCCGCCCCCAAAAGG-3′;

hHOTAIRdown 5′-CGCTTCAGCTGAGATGTTTGC-3′ and 5′-TGCTGTGGCCAGAATCCA-3′

hHOTAIRup 5′-ACACCACAGAGACACAAGCTCTAGAT-3′ and 5′-TATGGGTGCACATAAGAAGGAATATT-3′

hMALAT1prom 5′-CTGGGCGACAGAGCAAGAC-3′ and 5′-CACCTCTTTTTCTTGTTGTTGTTTTT-3′;

SCHLAP1prom 5′-CCTACTGGGAGGAACGAACAAC-3′ and 5′-TTCAGATGTGTTCGGAGTTTTTTC-3′.

### RNA-ChIP and RNA-Re-ChIP

RNA-Chromatin immunoprecipitation (n = 5) was performed with RNA-ChIP-IT (Active Motif) according manufacture’s instruction using specific antibodies to ERα and ERβ (Santa Cruz) or eNOS (BD transduction). Negative control was absence of antibody (NoAb). For RNA-Re-ChIP assays, complexes were recovered after first immunoprecipitation as described in ref. 18 and subjected to a second round of IP as for RNA-ChIPs. LncRNA recovery was analyzed using RNA-to-Ct one step (Applied Biosystems).

### ChIRP

Chromatin Isolation by RNA Purification (ChIRP, n = 5) was performed as described^26^, using antisense DNA oligos specific for LacZ and HOTAIR^26^ and MALAT1 (designed on www.singlemoleculefish.com according^26^ and listed in Supplementary Table S1). DNA fragments were recovered and analyzed by qPCR. Data, normalized to the corresponding DNA input control, and represented as percentage of Input.

### Transfections of gapmer lncRNA antisense oligos

The silencing of long non-coding RNAs was obtained using LNA longRNA GapmeRs (Exiqon, Vedbaek, Denmark). Cells were transfected (n = 5) in “reverse” mode by Lipofectamine RNAiMAX (Invitrogen), according to the manufacturer’s instructions, and after 48 h reseeded for final treatments. The effect of interference was evaluated after 5 days of interference. For OSCs, slices were transfected using 150 pmol of LNA longRNA GapmeRs FAM-labeled into 3 µl of Lipofectamine RNAiMAX (Life Technologies, Carlsbad, CA), in a 150 µl final volume of Opti-MEM (GIBCO-ThermoFisherScientific Waltham, US-MA). Gapmer sequences were as follows: LACZ ID: 436919-1 and 436919-2; HOTAIR ID 340939-1 and 340939-2 (LNA longRNA GapmeR- Exiqon), MALAT1 and Negative Control as in ref. 39.

### Organotypic Slices cultures (OSCs)

Radical prostatectomy specimens (7 from PCa and 2 from BPH) have been obtained immediately post-surgery and the cores of putative PCa areas (examined, histopathological evaluated and approved by the pathologist of the Università Cattolica, kept on ice cold and oxygenated cutting solution until slicing. The procedure was essentially according Stoppini *et al*. with minor modifications^40^. Briefly, tissue specimens have been cut into thick slices (350 μm) using McILWAIN TISSUE CHOPPER and cultured at a liquid-air interface using semi-porous tissue culture inserts (PICM03050, Millipore) placed in a six-well culture plate. Slices were cultured at 37 °C in humidified air with 5% CO2 with Iscove’s Modified Dulbecco’s Medium (IMDM) (GIBCO-ThermoFisherScientific Waltham, US-MA) supplemented with 10% hormone depleted serum FBS (HyClone), 2 mM glutamine, 100 µg/ml penicillin and streptomycin (method was optimized with regard to number of slices for tissue culture inserts, 5 slices each). Medium was replaced daily and after 3 or 4 days, slices were treated with or without E2, then removed from insert and transferred at −80° for final RNA/proteins extraction.

This study was authorized by the ethical committee of Fondazione Policlinico Gemelli-Università Cattolica in Rome, Italy (Protocol number: 25519/16; ID: 1247) and an informed consent was obtained from each patient. All procedures were conducted according to the principles expressed in the Declaration of Helsinki, the institutional regulation and Italian laws and guidelines.

### Statistical analysis

Data are expressed as mean ± SEM or as fold of induction as indicated in figures legend. Significance was calculated using a two-tailed t-test and/or one-way Analysis of Variance (ANOVA). Statistical analysis was performed using Sigma Plot 13.0 statistical software. *p* values of <0.05 were considered as significant in all tests.

## Additional Information

**How to cite this article**: Aiello, A. *et al*. MALAT1 and HOTAIR Long Non-Coding RNAs Play Opposite Role in Estrogen-Mediated Transcriptional Regulation in Prostate Cancer Cells. *Sci. Rep.* **6**, 38414; doi: 10.1038/srep38414 (2016).

**Publisher's note:** Springer Nature remains neutral with regard to jurisdictional claims in published maps and institutional affiliations.

## Supplementary Material

Supplementary Figures and Table

## Figures and Tables

**Table 1:** Clinical and Pathologic Feature of Patients and Tumors.

Patients	Age	PSA (ng/mL)	Gleason Score combined	Pathologic Stage
BPH	73	6,5	NA	NA
#1	66	11,98	7 (4 + 3)	pT2c pNx pMx
#2	58	5	7 (3 + 4)	pT2c pNx pMx
#3*	73	15,75	7 (3 + 4)	pT3a pNx pMx
#4	56	2,15	7 (4 + 3)	pT3a pNx pMx
#5	68	8,32	7 (4 + 3)	pT2c pNx pMx
#7	69	4,72	7 (4 + 3)	pT3b pNx pMx
#8	71	14	7 (3 + 4)	pT2c pNx pMx

^*^Controlateral hyperplastic tissue was included in Fig. 4b as BPH#3.
