# Supplementary material for: MALAT1 and HOTAIR Long Non-Coding RNAs Play Opposite Role in Estrogen-Mediated Transcriptional Regulation in Prostate Cancer Cells
Source: Sci Rep. 2016 Dec 6;6:38414. doi: 10.1038/srep38414 (PMC5138831; doi:10.1038/srep38414)
Supplement: Supplementary Figures and Table [file srep38414-s1.pdf]

MALAT1 AND HOTAIR LONG NON-CODING RNAS PLAY OPPOSITE ROLE IN ESTROGEN-MEDIATED TRANSCRIPTIONAL REGULATION IN PROSTATE CANCER CELLS

Aurora Aiello[1,2], Lorenza Bacci[2], Agnese Re[1], Cristian Ripoli[3], Francesco Pierconti[4], Francesco Pinto[5], Riccardo Masetti[6], Claudio Grassi[3,7], Carlo Gaetano[8], Pier Francesco Bassi[5], Alfredo Pontecorvi[2], Simona Nanni[2]*, Antonella Farsetti[1,9]*

[1]National Research Council, Institute of Cell Biology and Neurobiology, Rome, 00143, Italy
[2] Università Cattolica, Institute of Medical Pathology, Rome, 00168, Italy
[3] Università Cattolica, Institute of Human Physiology, Rome, 00168, Italy
[4] Università Cattolica, Institute of Pathology, Rome, 00168, Italy
[5] Università Cattolica, Fondazione Policlinico 'A. Gemelli', Urological Clinic, Rome, 00168, Italy
[6] Università Cattolica, Multidisciplinary Breast Center, Fondazione Policlinico 'A. Gemelli', Rome, 00168, Italy
[7] San Raffaele Pisana Scientific Institute for Research, Hospitalization and Health Care, 00163 Rome, Italy
[8] Goethe University Frankfurt, Division of Cardiovascular Epigenetics, Department of Cardiology, Internal Medicine Clinic III, Frankfurt am Main, 60590, Germany
[9] Goethe University Frankfurt, Internal Medicine Clinic III, Frankfurt am Main, 60590, Germany

*Corresponding authors:
Antonella Farsetti, MD
Institute of Cell Biology and Neurobiology,
National Research Council,
Rome, 00143, Italy
Ph. +39 0630154966
Email: antonella.farsetti@cnr.it
and
*Simona Nanni, PhD
Institute of Medical Pathology,
Università Cattolica di Roma
Rome, 00168, Italy
Ph. +39 0630154966
Email: simona.nanni@unicatt.it

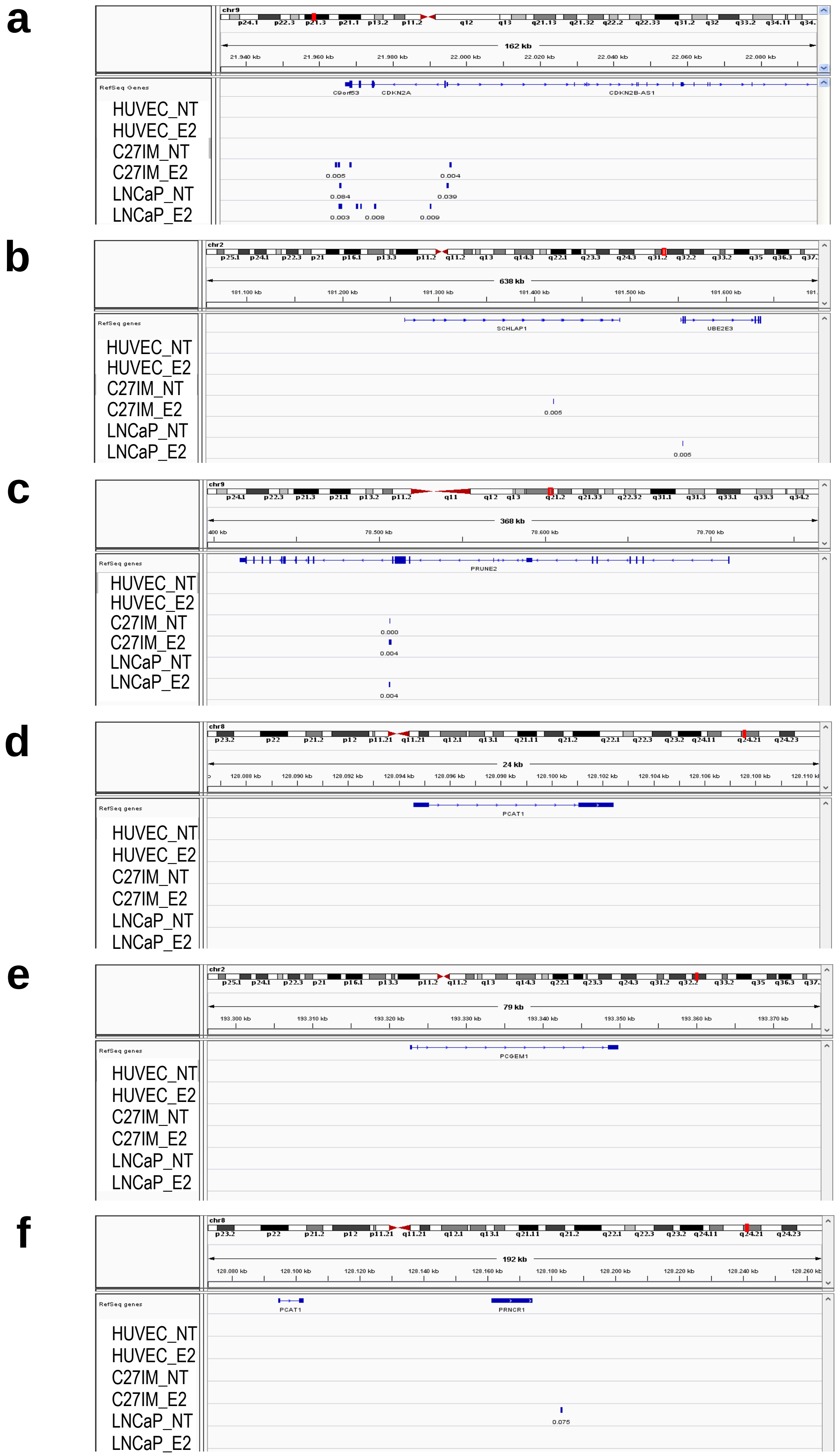

Supplementary Figure S1. Schematic representation of the peaks of eNOS by ChIP–Seq. Integrated Genome Viewer (IGV 2.3) screenshots showing peaks of eNOS identified by ChIP–Seq at the genomic regions encoding selected cancer-associated lncRNAs (a, b, c, d, e and f, CDKN2B-AS1 (also known as ANRIL) and SCHLAP1, PCA3, PCAT1, PCGEM1 and PRNCR1) in two prostate cancer cell lines, LNCaP and C27IM, and in the human endothelial cell line HUVEC, in the absence (NT) or presence of E2 (E2).

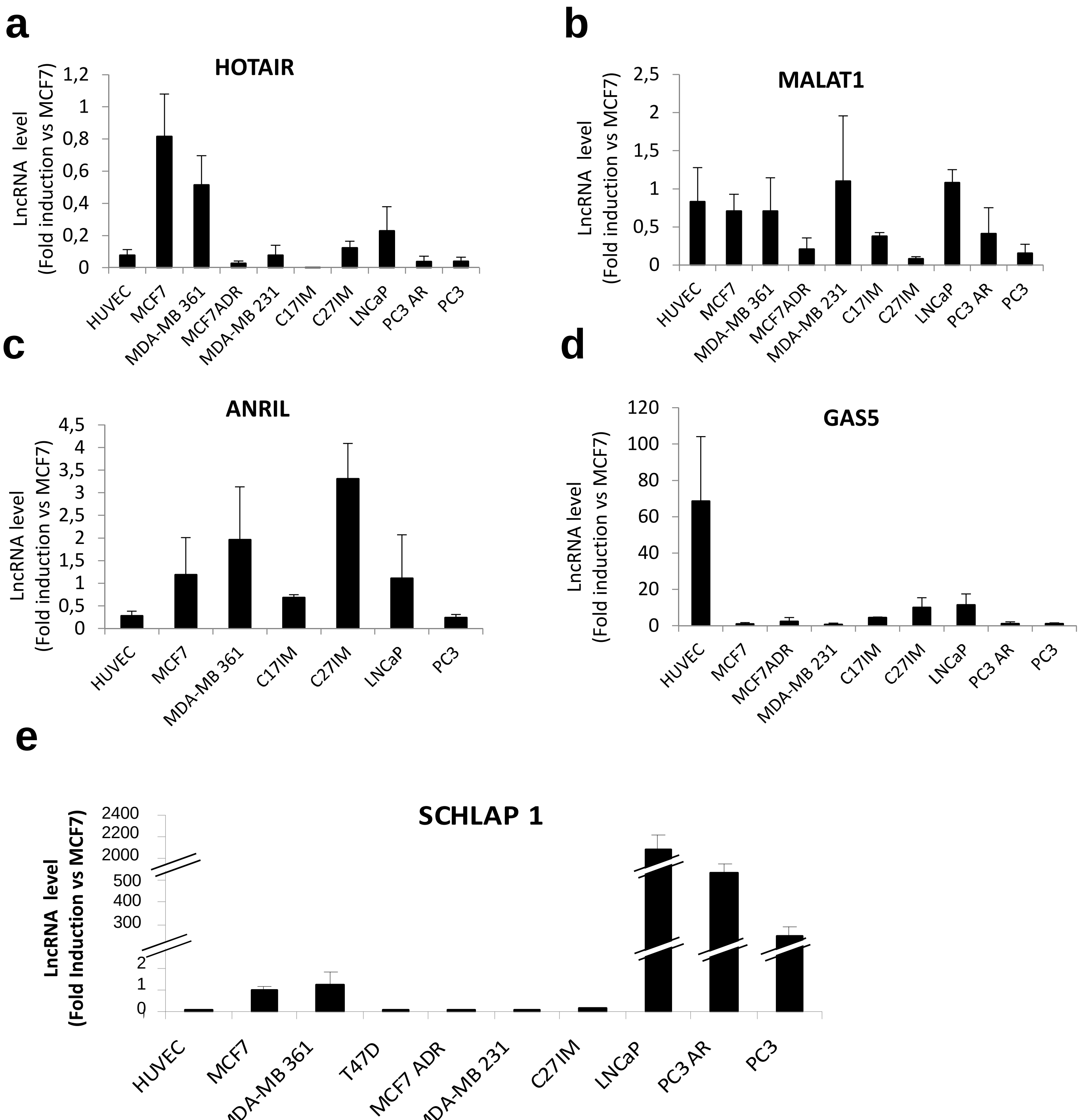

Supplementary Figure S2. Basal expression of LncRNAs cancer-associated in a variety of breast and prostate cancer cell lines. LncRNAs expression levels (HOTAIR (**a**), MALAT1 (**b**), ANRIL (**c**), GAS5 (**d**), and SCHLAP1 (**e**), were quantified by qRT-PCR in normal HUVEC, prostate hyperplastic C17IM, BCa (MCF-7, MDA-MB 361, MCF7-ADR and MDA-MB 231, T47D) and PCa (C27IM, LNCaP, PC3AR and PC3) cells in basal condition and represent the average+/-SEM of 5 independent experiments.

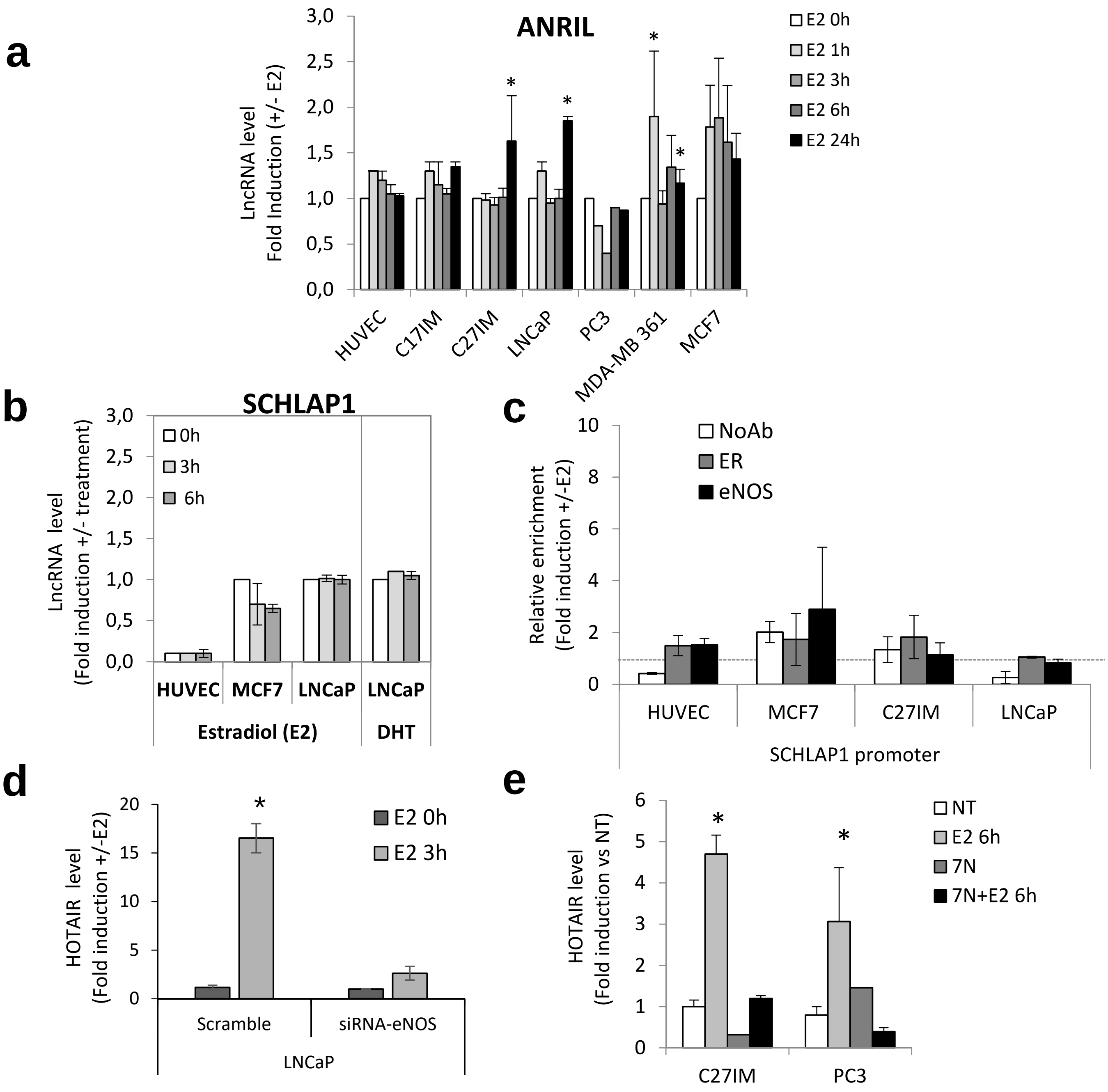

Supplementary Figure S3. Estrogen-dependent lncRNAs expression and eNOS/ERs recruitment in prostate and breast cancer cells and effect of eNOS interference on HOTAIR expression. a and **b**) Quantification of lncRNAs expression by qRT-PCR in normal HUVEC, prostate hyperplastic C17IM, BCa (MCF-7, MDA-MB 361) and Pca (C27IM, LNCaP, PC3) cells in basal condition (E2 0h) and after 1h, 3h, 6h and 24h of treatment with 10nM E2 (**a**) or after 3h and 6h of treatment with 10nM DHT (**b**). The results are plotted as fold induction (+/- treatment) and represent the average of 5 independent experiments. *p<0,05 vs E2 0h. **c**) Recruitment of Estrogen Receptors and eNOS, on the promoter region of SCHLAP1 by ChIPs in the presence or absence of E2 in HUVEC, breast (MCF7) and prostate (C27IM and LNCaP) cells. The immunoprecipitations were performed using anti ERα (in HUVEC and MCF-7), ERβ (in C27IM and LNCaP) and eNOS or no antibody (NoAb) as a negative control. Values are represented as Fold of Induction (+/- E2) and as mean +/-SEM of 4 independent experiments. **d)** HOTAIR level in LNCaP cells before and after eNOS interference (siRNA-eNOS) in presence or absence of estradiol (E2). Scramble served as negative control. The results are plotted as fold induction (+/- E2) and represent the average of 4 independent experiments (* p <0,05 vs E2 0h). **e**) HOTAIR levels are quantified by qRT-PCR in C27IM and PC3 cells in basal condition (NT) or upon E2 and/or before/after treatment with 7Nitroindazole (7N). The results are plotted as fold induction vs NT and represent the average of 3 independent experiments (* p <0,05 vs NT).

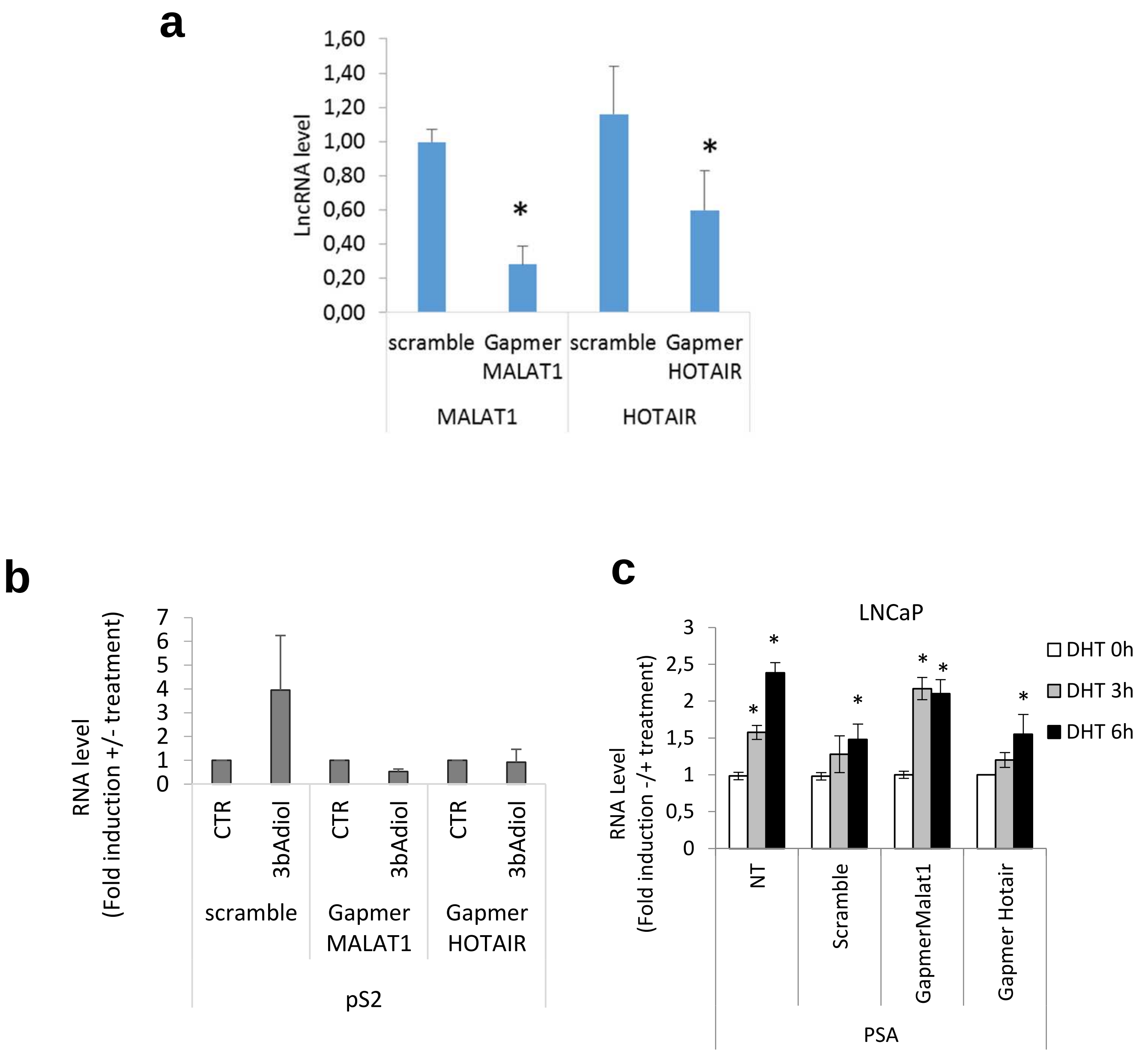

Supplementary Figure S4. Interference for HOTAIR and MALAT1 by Gapmers antisense oligonucleotides and effect on sex-steroid hormone responsiveness.

a) Levels of lncRNAs quantified by qRT-PCR in PCa cells transfected with specific or scramble gapmer. The results are plotted as fold induction vs scramble. *****p<0,05 Gapmer vs Scramble. **b**) pS2 level in PCa cells transfected with specific or scramble gapmers in basal condition (CTR) and after 10nM 3b-Adiol treatment of 6 hours. Results are plotted as fold induction +/- treatment. *p <0.05 3bAdiol vs CTR. **c**) PSA level in LNCaP mock-transfected (NT) or transfected with MALAT1, HOTAIR or scramble gapmers before (0h) and after 10 nM DHT treatment of 3 or 6 hours. The results are plotted as fold induction as in +/- treatment. *p <0.05 vs 0h.

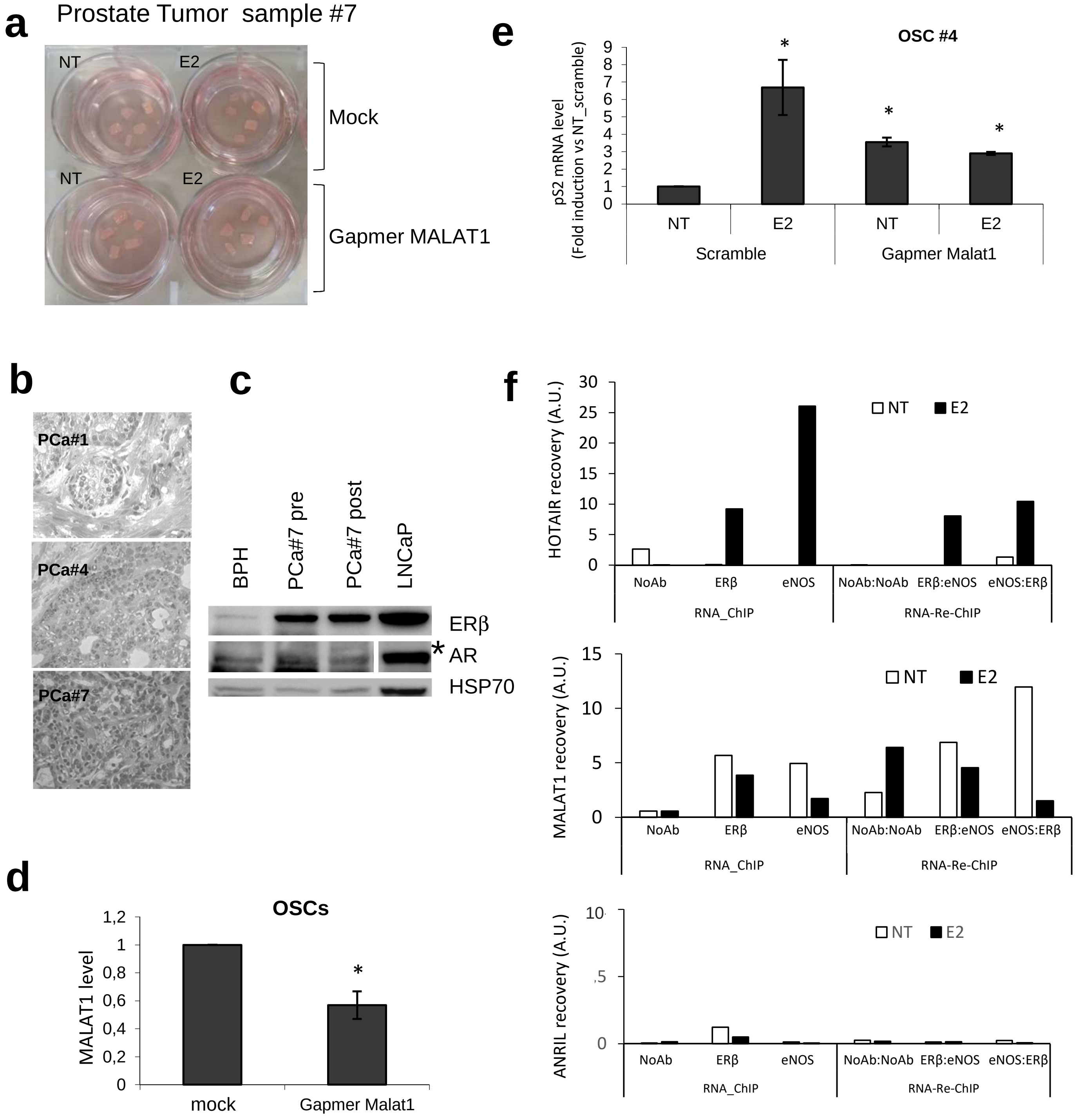

Supplementary Figure S5. Organotypic slice cultures of Prostate Tumors, *in vivo* interference for MALAT1 and analysis by RNA-Re-ChIP of lncRNA and ER/eNOS complex. a) Representative Organotypic Slices (OSCs) after 5 days with or without Gapmer_MALAT1. OSCs were treated with 17β-estradiol for 6h before harvesting. Control is OSC not-estradiol-treated, mock-transfected (NT). **b**) Hematoxylin and eosin staining of tissue counterpart before OSCs preparation. **c**) Analysis of ERβ and AR by Western blotting on total extracts from prostate tissue of BPH, PCa#7 (freshly prepared, pre) and after 5 days in dish as OSCs (post) as in **a**. LNCaP served as positive control (*lower exposure). HSP70 was used as loading control. **d**) Levels of MALAT1 quantified by qRT-PCR in OSC transfected with specific MALAT1 Gapmer. The results are plotted as fold induction vs mock. ***p<0,05 Gapmer vs mock. **e**) mRNA levels in OSC#4 quantified by qRT-PCR in PCa cells transfected with MALAT1 gapmers and treated with or without 17β-estradiol. The results are plotted as fold induction vs “NT” +/- SEM. *p <0.05 vs NT. **f**) *In vivo* analysis of HOTAIR, MALAT1 or ANRIL interaction with ERβ/eNOS complex before and after estrogen treatment detected by RNA-Re-ChIP (Representative experiment).

Supplemetary Table S1. ChIRP oligos

Probe Name	Sequence (5' to 3')	Even	Odd
MALAT1_1	caaggactctgggaaacctg		x
MALAT1_2	aggacagctaagatagcagc	x	
MALAT1_3	ctaaataccaccacctggaa		x
MALAT1_4	acacccagaagtgtttacac	x	
MALAT1_5	gaagacacagagaccttggg		x
MALAT1_6	ctaagcgaatggctttgtct	x	
MALAT1_7	caaggcaaatcgccatggaa		x
MALAT1_8	tggcaaaatggcggactttc	x	
MALAT1_9	gtgatagttcagggctttac		x
MALAT1_10	aggtatagtttaccaccttt	x	
MALAT1_11	cctcttaaagcacttcttgt		x
MALAT1_12	catcaccggaattcgatcac	x	
MALAT1_13	gcgaggcgtatttatagacg		x
MALAT1_14	ctcccaattaatctttccat	x	
MALAT1_15	tctccaaattgtttcatcct		x
MALAT1_16	atcttctcaagctttacctt	x	
MALAT1_17	tacttccgttacgaaagtcc		x
MALAT1_18	ctgggtcagctgtcaattaa	x	
MALAT1_19	tcagtcctagcttcatcaaa		x
MALAT1_20	aacaacatattgccgacctc	x	
MALAT1_21	agtcatttgcctttaggatt		x
MALAT1_22	aactgtaaacctgtggtggt	x	
MALAT1_23	ccaaggataaaagcagctcc		x
MALAT1_24	tgaaccaaagctgcactgtg	x	
MALAT1_25	actgccaactaattgccaat		x
MALAT1_26	ccagtggctcatatttaact	x	
MALAT1_27	actttccttgcccaaattaa		x
MALAT1_28	cccaatggaggtatgacata	x	
MALAT1_29	atctctcatttatttcggct		x
MALAT1_30	gatacctgtctgaggcaaac	x	
MALAT1_31	tgaagtgtactatcccatca		x
MALAT1_32	tctttcctgccttaaagtta	x	
MALAT1_33	tgtcaatttatagacccctg		x
MALAT1_34	aaacattgcctaccactcta	x	
MALAT1_35	cctgaatggcttcatgaagg		x
MALAT1_36	tgcatttacttgccaacaga	x	
MALAT1_37	gtcgtttcacaatgcattct		x
MALAT1_38	caacactcagcctttatcac	x	

MALAT1_39	ttttttcttactgggtctgg		x
MALAT1_40	ccactggtgaattcaactgg	x	
MALAT1_41	ttgtcccataactgatctga		x
MALAT1_42	aacacagtttgctcacatgc	x	
MALAT1_43	tgacacttctcttgacctta		x
MALAT1_44	cactccagaaagagggagtt	x	
MALAT1_45	agatcaaaaggcacggggtg		x
MALAT1_46	catcgttaccttgaaaccga	x	
MALAT1_47	taacatagttcaacccacca		x
MALAT1_48	ttgcaggcaaattaatggcc	x	
MALAT1_49	agttggttttagtcactgga		x
MALAT1_50	tatttctccacttactggtt	x	
MALAT1_51	gtctacaagttacatgttcc		x
MALAT1_52	cactcaaatgcctatcttct	x	
MALAT1_53	cacctgaaaaagtcttagca		x
MALAT1_54	tagcttggccaagtctgtta	x	
MALAT1_55	agatcagcttccgctaagat		x
MALAT1_56	tgaccctactgaagagcatt	x	
MALAT1_57	tggaaaccaggagtgccaac		x
MALAT1_58	gtagtcaaagcaaagacgcc	x	
MALAT1_59	cagaaagagtcctgaagaca		x
MALAT1_60	ccccaaaactccaagaacta	x	
MALAT1_61	agatattgtgctgttacctc		x
MALAT1_62	tcaagtcaagctcctgacaa	x	
MALAT1_63	ggcagttatattaggttctc		x
MALAT1_64	ctgcaggctattacctgaaa	x	
MALAT1_65	agcagtagggcttctcaaaa		x

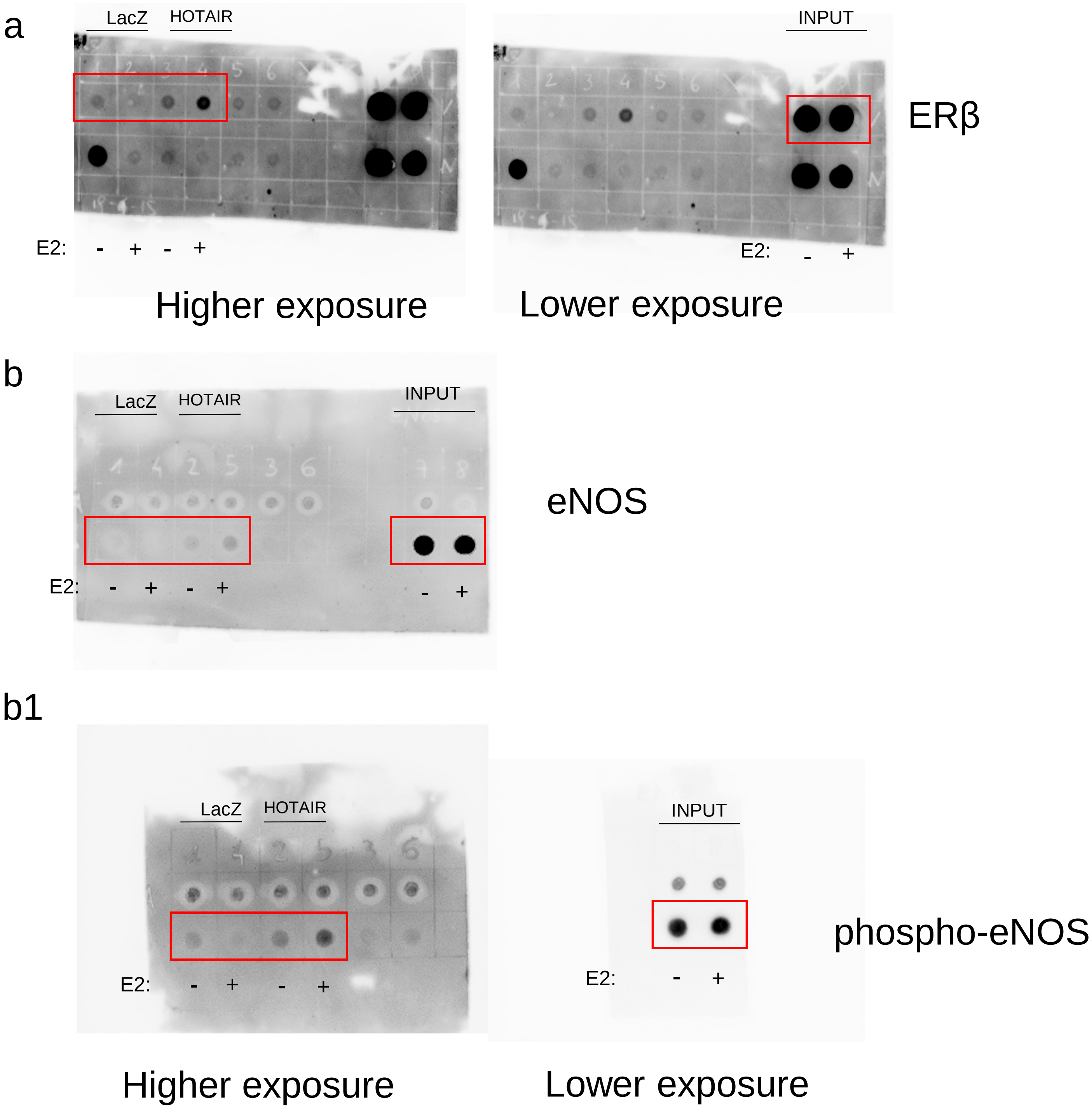

Supplementary raw data for Fig. 2d.

ERβ (a) and eNOS (b) were blotted on parallel membranes. eNOS membrane was stripped and reblotted for phospho-eNOS (b1). Red Boxes indicate the selected non-contiguous lanes and different exposures showed in Fig. 2d. Dot Blot for phospho-eNOS was exposed after cutting the membrane to avoid problem derived from very different exposition time required.
